# Highly Efficient and Durable Ammonia Electrolysis Cell Using Zirfon Separator

**DOI:** 10.1002/advs.202500579

**Published:** 2025-01-31

**Authors:** Haeyong Shin, Sang‐Mun Jung, Young Jin Lim, O‐Jung Yim, Byung‐Jo Lee, Kyu‐Su Kim, In‐Ho Baek, Jinwoo Baek, Jinhyeon Lee, Yong‐Tae Kim

**Affiliations:** ^1^ Department of Materials Science and Engineering Pohang University of Science and Technology Gyeongbuk 37673 Republic of Korea

**Keywords:** ammonia, ammonia electrolysis cell, ion conductivity, operating conditions, zirfon

## Abstract

Most studies on ammonia electrolysis have focused on anion exchange membranes (AEMs), which face limitations in operating conditions, such as pH and ammonia concentration. This study introduces a novel concept of an ammonia electrolysis cell (AEC) utilizing a Zirfon separator capable of operating under high pH and ammonia concentrations. The Zirfon‐based AECs achieve a peak current density of 915 mA cm^−2^, representing the highest reported value in AEC literature. Additionally, the Zirfon separator exhibits less conductivity degradation than AEMs during cycling tests (Zirfon 14.1%, AEMs 30.2%), demonstrating superior durability of the Zirfon‐based AEC.

## Graphical abstract

This study introduces a novel Zirfon‐based ammonia electrolysis cell (AEC) using a Zirfon separator, demonstrating superior performance under high pH and ammonia concentrations. The cell achieves a peak current density of 915 mA cm⁻^2^, the highest reported in AEC literature, with enhanced durability and lower conductivity degradation compared to traditional AEM‐based AECs.

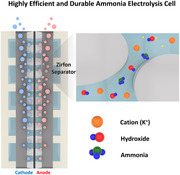

## 1 Introduction

Hydrogen has significant potential for conversion into diverse chemicals and environmentally friendly production.^[^ ^1^ ^]^ However, the efficient, economical, and safe handling and transport of hydrogen in large quantities presents major challenges to its utilization.^[^ ^2^ ^]^ To address these challenges and advance the hydrogen‐based economy concept various hydrogen carriers have been proposed, including liquid hydrogen, methylcyclohexane (MCH), and ammonia.^[^ ^3^ ^]^ Liquid hydrogen can be used directly without conversion; however, the liquefaction process^[^ ^4^ ^]^ results in approximately 45% energy loss. In contrast, MCH can be converted to toluene at room temperature, eliminating the need for cooling. Nonetheless, MCH has a low hydrogen uptake (about 3%) and requires high‐temperature heat for hydrogen extraction.^[^ ^3^ ^]^ Due to the limitations of other hydrogen carriers, ammonia stands out as a superior option for hydrogen storage. It is cost‐effective, has extensive infrastructure, boasts a high volumetric density (1.7 times that of H_2_), and can be liquefied at relatively low pressure (≈8 bar). Additionally, its decomposition does not produce harmful substances like NO_x_ or CO_x_, making it the most promising candidate.^[^ ^3^ ^]^

The traditional method for large‐scale hydrogen extraction from ammonia is thermal catalytic cracking. This technique efficiently decomposes substantial ammonia but typically operates at elevated temperatures (>400 °C) due to limited activity at lower temperatures.^[^ ^5^ ^]^ Additionally, residual ammonia in the extracted hydrogen requires further processing through methods such as temperature swing adsorption and pressure swing adsorption for purification.^[^ ^6^ ^]^ As an alternative to thermochemical methods, electrochemical catalytic cracking, known as ammonia electrolysis, offers a viable option. It has a theoretical cell voltage of 0.06 V, making it approximately 95% less energy‐intensive than water electrolysis^[^ ^7^ ^]^ facilitating straightforward hydrogen extraction without relying on supplementary auxiliary facilities and reducing operational costs.

However, the low efficiency of ammonia electrolysis cells (AECs) remains a significant barrier to commercialization.^[^ ^8^ ^]^ During ammonia electrolysis, the hydrogen evolution reaction (HER) and ammonia oxidation reaction (AOR) occur simultaneously (Equations (1)–(3)):^[^ ^9^ ^]^

(1) $$\begin{array}{ccc} \mathrm{AOR}:2{\mathrm{NH}}_{3}\left(\mathrm{aq}\right) & & +6{\mathrm{OH}}^{-}\to N_{2}\left(g\right)+6H_{2}O\left(l\right)\\ & & +6e^{-}\left(E^{0}=-0.77\;V\;\mathrm{vs}.\;\mathrm{SHE}\right) \end{array}$$

(2) $$\begin{array}{ccc} \mathrm{HER}:6H_{2}O\left(l\right) & & +6e^{-}\to 3H_{2}\left(g\right)\\ & & +6{\mathrm{OH}}^{-}\left(E^{0}=-0.83\;V\;\mathrm{vs}.\;\mathrm{SHE}\right) \end{array}$$

(3) $$\mathrm{Overall}:2{\mathrm{NH}}_{3}\left(\mathrm{aq}\right)\to 3H_{2}\left(g\right)+N_{2}\left(g\right)\left(E^{0}=0.06\;V_{\mathrm{cell}}\right)$$

Despite the rapid kinetics of the HER, the AOR poses a significant limitation due to the slow kinetics of its six‐electron transfer process. Consequently, most research on ammonia electrolysis has focused on enhancing the efficiency of AOR catalysts;^[^ ^10^ ^]^ however, practical demonstrations in actual AECs, approached systematically remain limited.

Traditionally, AECs have predominantly relied on alkaline electrolysis, employing two electrode plates separated by a liquid alkaline electrolyte.^[^ ^10^, ^11^ ^]^ However, this configuration results in limited hydrogen conversion due to high solution resistance and the inability to stack cells. Recognizing these limitations, recent literature on ammonia electrolysis has shifted toward anion exchange membrane (AEM)‐based AECs, which enable minimizing the interelectrode gap (<2 mm) and stacking cells to achieve higher H_2_ production rates.^[^ ^10^, ^12^ ^]^ Despite these advantages, AEM‐based AECs still face fundamental challenges, including low ionic conductivity,^[^ ^13^ ^]^ limited chemical stability under high pH,^[^ ^14^ ^]^ and susceptibility to NH_3_ poisoning.^[^ ^15^ ^]^

This study presents a novel AEC utilizing a Zirfon separator capable of operating under high pH and ammonia concentrations. This novel cell combines the advantages of traditional alkaline electrolysis, which is optimal for high pH environments, with the stackable features of AEM‐based electrolysis.^[^ ^16^ ^]^ By employing a Zirfon‐based AEC in these conditions, we achieved a peak current density of 915 mA cm^−2^ at 1 V, the highest value reported in the AEC literature. In terms of durability, after 100 repeated polarization curves, we demonstrated that the reduction in ion conductivity for Zirfon (14.1%) was significantly lower than that for AEM (30.2%). Furthermore, additional durability tests conducted at a fixed current density revealed that the Zirfon‐based AEC maintained stable operation for over 18 hours, whereas the AEM‐based AEC exhibited a rapid performance degradation after 70 minutes, indicating the Superior performance of the Zirfon‐based AEC.

## 2 Results and Discussion

### 2.1 Zirfon‐Based AEC Configuration

**Figure 1:**
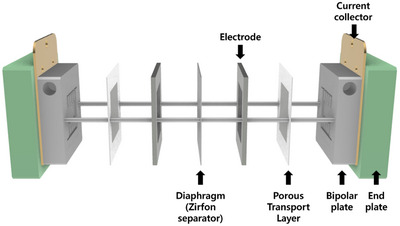
Cell configurations for the Zirfon‐based AEC.

**Figure** **1** illustrates a schematic representation of the Zirfon‐based AEC. This configuration includes various components: the anode and cathode electrodes, a diaphragm (acting as a gas separator), porous transport layers (PTLs), bipolar plates, cell frames, gaskets, and endplates. The Zirfon‐based AEC design, similar to the configuration used in AEM‐based AEC, refers to a system where two porous electrodes are compressed on either side of a hydroxide ion‐conducting membrane or separator—in this case, the Zirfon separator used in our study. This design can minimize the gap between the two electrodes to be equal to the thickness of the separator (≈0.5 mm) while operating under high pH conditions. The Zirfon separator (UTP 500+, Agfa, 0.5 mm thickness), which consists of an open mesh polyphenylene sulfide fabric symmetrically coated with a mixture of a polymer and zirconium oxide, frequently used as the diaphragm. During ammonia electrolysis, it serves as an electrical insulator and facilitates the transport of hydroxide ions while effectively separating hydrogen and nitrogen gases. Although it may lead to some level of H_2_ and N_2_ crossover compared to AEMs, it has been successfully used in MW‐scale alkaline water electrolyzers (AWEs) due to its robust performance in harsh conditions, including 30 wt.% KOH solutions at 80 °C.^[^ ^17^ ^]^ PTLs ensure an even distribution of reactants and direct the heat generated by the electrochemical reaction to the bipolar plate. The bipolar plate then distributes the electrolyte uniformly via the external manifold. The cell frame, connected to the PTL periphery, supplies electrolytes to the individual cells through designated channels. The gasket prevents gas or liquid leakage at the contact interfaces. Once assembled, these components are compressed together using the endplate. The operation and components of the cell are depicted in Figures S1–S3 (Supporting Information).

### 2.2 Zirfon‐Based AEC Assembly

Initially, we selected the electrode materials for the anode to evaluate the performance of the AEC. We assessed the AEC performance using Pt/C as the anode and compared it with two other carbon‐supported metals: iridium (Ir/C) and palladium (Pd/C). Pt/C was used as the cathode electrode across all scenarios for comparison purposes. Nanoparticles were uniformly dispersed on the Ni foam‐based PTL electrode (Figure S4, Supporting Information). As shown in Figure S5 (Supporting Information), Ir/C demonstrated a lower onset potential (0.4 V) compared to Pt/C (0.5 V) and Pd/C (0.9 V), indicating an earlier initiation of ammonia dehydrogenation.^[^ ^10^, ^18^ ^]^ Although Pt/C exhibited a slightly higher onset potential than Ir/C, its enhanced dehydrogenation and lower nitrogen affinity led to superior AOR performance, with a peak potential (V_peak_) of 0.75 V, and a peak current density (j_peak_) of 200 mA cm^−2^, significantly higher than Ir/C (V_peak_ = 0.55 V, j_peak_ = 102.5 mA cm^−2^) and Pd/C (V_peak_ = 1 V, j_peak_ = 37.5 mA cm^−2^).^[^ ^19^ ^]^ To exclude the possibility of the oxygen evolution reaction (OER), the voltage range was set between 0 and 1 V (Figure S6, Supporting Information). Figure S7 (Supporting Information) revealed that the high current density is entirely due to the AOR. Due to the strong affinity of Pd for OH^−^ adsorption, Pd/C showed reduced AEC performance than Ir/C and Pt/C.^[^ ^20^ ^]^ Consequently, Pt/C was selected as the anode catalyst for the Zirfon‐based AEC.

**Figure 2:**
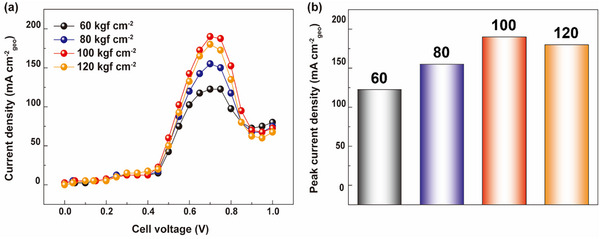
a) AEC polarization curves and b) peak current densities at different clamping pressures. The optimal clamping pressure for our AEC system is 100 kgf cm^−2^.

To enhance the performance of electrochemical systems, it is essential to optimize both the cell‐level configurations and the assembly process, as well as to employ highly efficient electrocatalysts. A key factor in cell assembly is the clamping pressure, which can significantly influence the performance of electrolyzers.^[^ ^21^ ^]^ To investigate this, we conducted performance tests on AECs with clamping pressures ranging from 60 to 120 kgf cm^−2^ (**Figure** **2**a). These experiments utilized an electrolyte of 8 m KOH and 1 m NH_3_ at 70 °C, with a flow rate of 200 cc/min. We observed a steady improvement in AEC performance with increasing clamping pressure, as the j_peak_ rose from 122.5 mA cm^−2^ at 60 kgf cm^−2^ to 190 mA cm^−2^ at 100 kgf cm^−2^ (Figure 2b). However, performance began to decline beyond 100 kgf cm^−2^. This trend can be attributed to changes in the resistance of the electrolytic cell with varying clamping pressures. According to Phillips et al., the resistance of the electrolytic cell can be expressed by Equation (4):^[^ ^16a^ ^]^

(4) $$R_{\mathrm{cell}}=R_{\mathrm{circuit}}+R_{\mathrm{electrolyte}}+R_{\mathrm{bubbles}}+R_{\mathrm{contact}}$$

Among these components, clamping pressure directly affects R_electrolyte_ and R_contact_. Increasing the clamping pressure reduces electrode spacing, thereby decreasing R_electrolyte_.^[^ ^16a^ ^]^ Additionally, higher clamping pressure decreases the contact resistance (R_contact_) between cell components, enhancing AEC performance. However, excessive clamping pressure leads to gas bubble accumulation, which impedes gas release and obstructs active electrode sites, resulting in decreased performance.^[^ ^22^ ^]^ This decline in current density values at clamping pressures above 100 kgf cm^−2^ confirms this effect. Consequently, the optimal clamping pressure for our AEC system is 100 kgf cm^−2^.

### 2.3 Cell Operating Condition (1): Flow Rate

**Figure 3:**
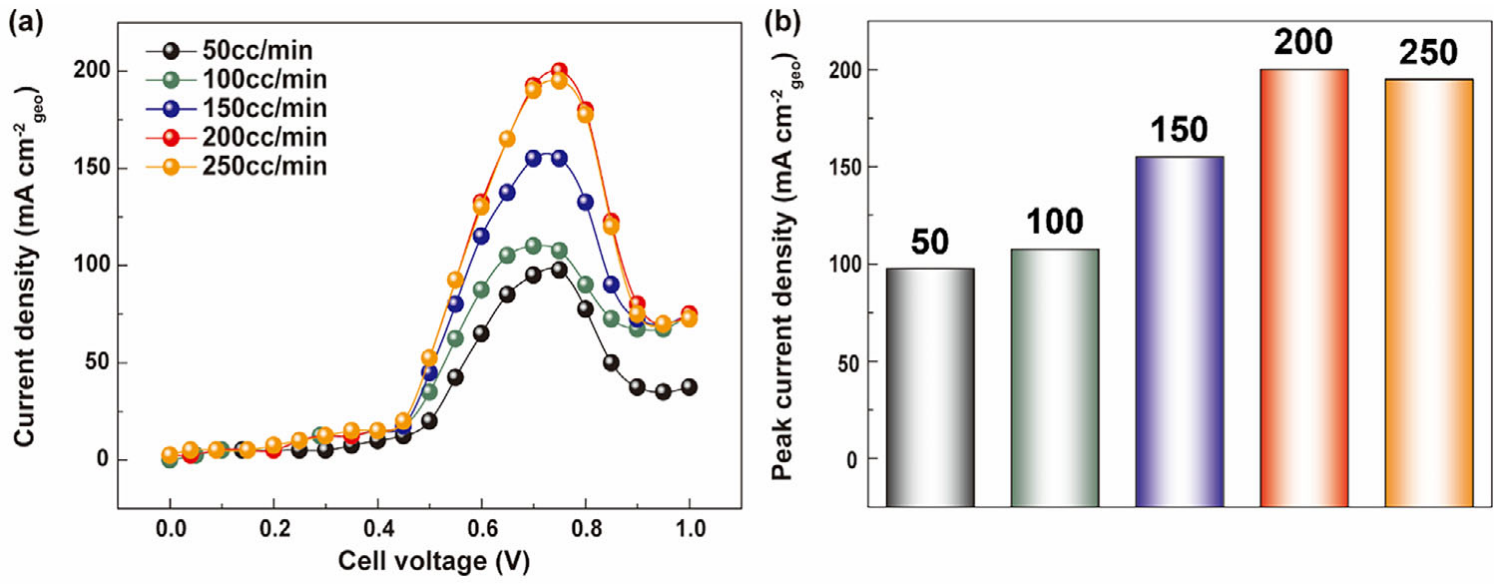
a) AEC polarization curves and b) peak current densities at different flow rates. The optimal flow rate for our AEC system is 200 cc/min.

For the ammonia conversion to hydrogen, efficient mass transport is crucial to deliver ammonia to the electrode, making rapid ammonia transport a key parameter. Typically, mass transport occurs through two main mechanisms: (1) convection and (2) diffusion based on concentration gradients (Fickian diffusion).^[^ ^23^ ^]^ Forced convection, facilitated by the flow of electrolytes, can be an effective transport method in AECs. To evaluate the impact of electrolyte flow rate on the current density of the AEC, we conducted performance tests with flow rates ranging from 50 to 250 cc/min (**Figure** **3**a). These experiments used an 8 m KOH + 1 m NH_3_ electrolyte at 70 °C and a clamping pressure of 100 kgf cm^−2^. As depicted in Figure 3b, the j_peak_ of the cell increased with higher electrolyte flow rates, rising from 110 mA cm^−2^ at 50 cc/min to 200 mA cm^−2^ at 200 cc/min. This indicates that mass transport predominantly influences cell performance. Beyond a flow rate of 200 cc/min, no significant change was observed (j_peak_ = 195 mA cm^−2^), suggesting that the primary determinant of cell performance shifts from mass transport to kinetics once a sufficient flow rate is achieved. Therefore, the optimal flow rate for our AEC system was determined to be 200 cc/min.

### 2.4 Cell Operating Condition (2): Electrolyte Concentration

Increasing the electrolyte concentration can reduce cell resistance and boost ammonia conversion rates in AECs, as suggested by the Butler‐Volmer equation. To explore this relationship, we examined the performance of AECs under varying electrolyte concentrations. Ammonia electrolysis typically occurs in alkaline conditions. In low pH environments, NH_3_ reacts with acids, leading to neutralization and the formation of NH_4_^+^ instead of NH_3_, which hinders hydrogen production.^[^ ^24^ ^]^ Thus, the ammonia electrolysis is more effective in an alkaline media. For electrolyte concentration in AECs, two factors are considered: 1) hydroxide ion (OH^−^) concentration and 2) NH_3_ concentration.

**Figure 4:**
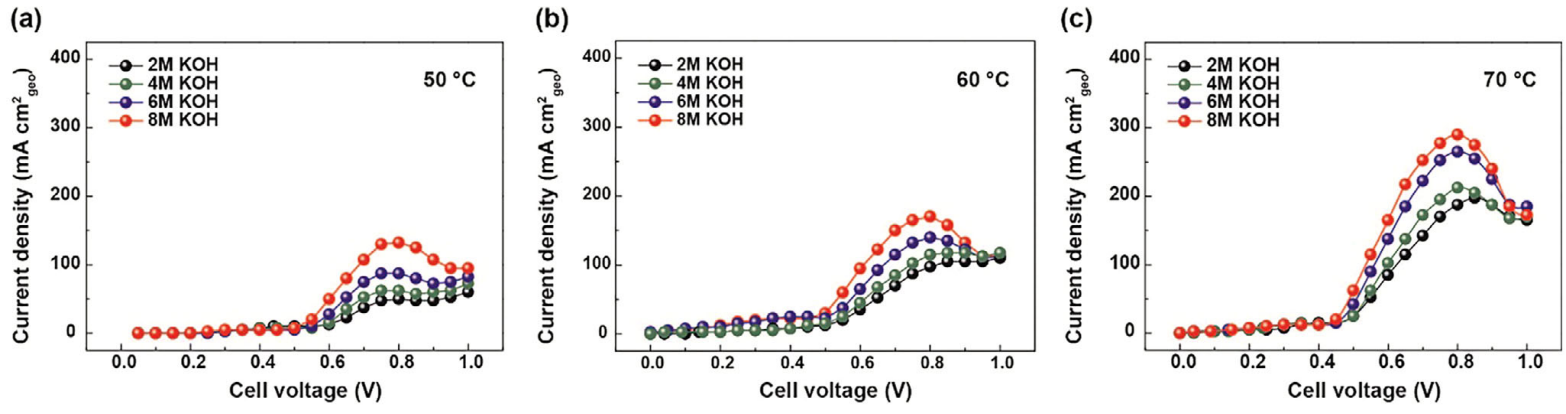
AEC polarization curves under 1 m NH_3_ and varying KOH concentrations at a) 50 °C, b) 60 °C, and c) 70 °C. Cell performance is directly proportional to KOH concentration.

Firstly, the OH^−^ concentration significantly affects AEC performance. A higher OH⁻ concentration in alkaline electrolytes can decrease ohmic resistance. However, an increased OH^−^ concentration might also raise electrolyte viscosity, potentially increasing mass transport resistance.^[^ ^25^ ^]^ Additionally, OH^−^ ions play a role in NH_3_ dehydrogenation during the AOR, potentially enhancing reaction kinetics (Equation (1)). Nonetheless, an excessively high OH^−^ concentration might block active NH_3_ sites on the electrode surface, reducing the AOR reaction rate.^[^ ^20^ ^]^ Therefore, evaluating the impact of KOH concentration on the AEC current density is essential. **Figure** **4** illustrates the performance data of AECs at different KOH concentrations at 50, 60, and 70 °C. For a consistent comparison, the ammonia concentration was maintained at 1 m. The flow rate and cell clamping pressure were set at 200 cc/min and 100 kgf cm^−2^, respectively. As depicted in Figure 4a, at 50 °C, the j_peak_ of the cell increased with higher KOH concentrations, from 50 mA cm^−2^ at 2 m KOH to 132.5 mA cm^−2^ at 8 m KOH. Similarly, at 60 and 70 °C, the j_peak_ increased from 105 mA cm^−2^ at 2 m KOH to 170 mA cm^−2^ at 8 m KOH for 60 °C, and from 187.5 mA cm^−2^ at 2 m KOH to 290 mA cm^−2^ at 8 m KOH for 70 °C (Figure 4b,c). Cell performance was directly proportional to KOH concentration at both temperatures, indicating that the reduction in ohmic resistance and the enhancement of dehydrogenation outweighed the potential increase in viscosity and obstruction of active sites.

**Figure 5:**
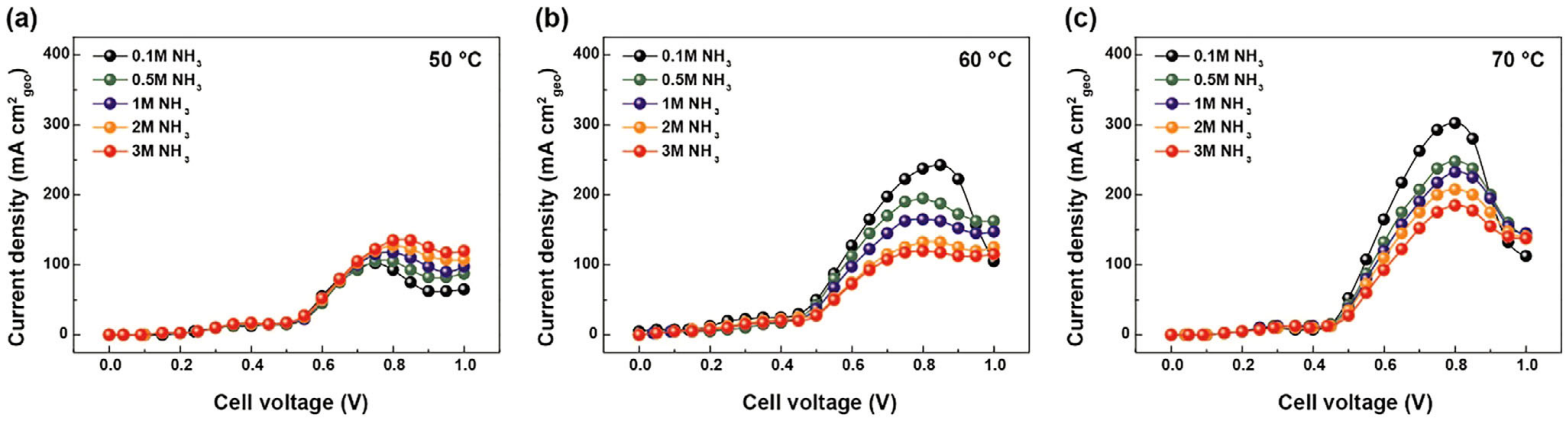
AEC Polarization curves under 8 m KOH and varying NH_3_ concentrations at a) 50 °C, b) 60 °C, and c) 70 °C.

Ammonia concentration significantly influences AEC performance. According to the Butler‐Volmer equation, increasing the NH_3_ concentration can enhance the ammonia conversion rates. Interestingly, the effect of ammonia concentration on cell performance demonstrated contrasting trends at 50, 60, and 70 °C, differing from the patterns observed for KOH concentration. At 50 °C, there was a direct correlation between ammonia concentration and cell performance, indicating that higher NH_3_ concentrations facilitate the AOR, as shown in **Figure** **5**a. However, at 60 and 70 °C, an inverse relationship was observed, where increased reactant concentration led to decreased cell performance, implying a potential shift in the reaction mechanism, as depicted in Figure 5b,c.

The mechanism of ammonia oxidation to nitrogen remains debated. Typically, the dimerization step can be classified into two primary mechanisms: the Gerischer–Mauerer (G–M) mechanism (Equations (5)–(10)), and Oswin–Salomon (O–S) (Equations (11)–(16)).^[^ ^26^ ^]^

[Gerischer and Mauerer (G–M) mechanism]

(5) $$\left(1\right)NH_{3}+OH^{-}+*\to NH_{2}*+H_{2}O+e^{-}$$

(6) $$\left(2\right)NH_{2}*+NH_{2}*\to N_{2}H_{4}*\left(\mathrm{Dimerization}\mathrm{step}\right)$$

(7) $$\left(3\right)N_{2}H_{4}*+OH^{-}\to N_{2}H_{3}*+H_{2}O+e^{-}$$

(8) $$\left(4\right)N_{2}H_{3}*+OH^{-}\to N_{2}H_{2}*+H_{2}O+e^{-}$$

(9) $$\left(5\right)N_{2}H_{2}*+OH^{-}\to N_{2}H*+H_{2}O+e^{-}$$

(10) $$\left(6\right)N_{2}H*+OH^{-}\to N_{2}+H_{2}O+*+e^{-}$$

[Oswin–Salomon (O–S) mechanism]

(11) $$\left(1\right)NH_{3}+OH^{-}+*\to NH_{2}*+H_{2}O+e^{-}$$

(12) $$\left(2\right)NH_{2}*+OH^{-}\to \mathrm{NH}*+H_{2}O+e^{-}$$

(13) $$\left(3\right)\mathrm{NH}*+OH^{-}\to N*+H_{2}O+e^{-}$$

(14) $$\left(4\right)N*+NH_{2}*\to N_{2}H_{2}*\left(\mathrm{Dimerization}\mathrm{step}\right)$$

(15) $$\left(5\right)N_{2}H_{2}*+OH^{-}\to N_{2}H*+H_{2}O+e^{-}$$

(16) $$\left(6\right)N_{2}H*+OH^{-}\to N_{2}+H_{2}O+*+e^{-}$$

As the operating temperature rises, the deprotonation of active NH_2_ species becomes more facile,^[^ ^27^ ^]^ potentially leading to an increase in inactive N* or NH* species that obstruct the active surface. This indicates a shift in the reaction mechanism from G–M to O–S. Prior DFT calculations^[^ ^27^ ^]^ have shown that the dimerization of N* + NH_2_* in the O–S mechanism is more challenging than the dimerization of two NH_2_* species in the G–M mechanism.^[^ ^26^ ^]^ Consequently, this leads to an accumulation of N* or NH* intermediates.^[^ ^27^ ^]^ The inverse trend observed in **Figure** 5b,c can be attributed to the high concentration of NH_3_ in the electrolyte, which accelerates the formation of N* or NH* radicals. Thus, we confirm that the reaction mechanism shifts from G–M to O–S under relatively high‐temperature conditions, and an increase in NH_3_ concentration at elevated temperatures can hinder catalytic activity.

### 2.5 Cell Operating Condition (3): Temperature

**Figure 6:**
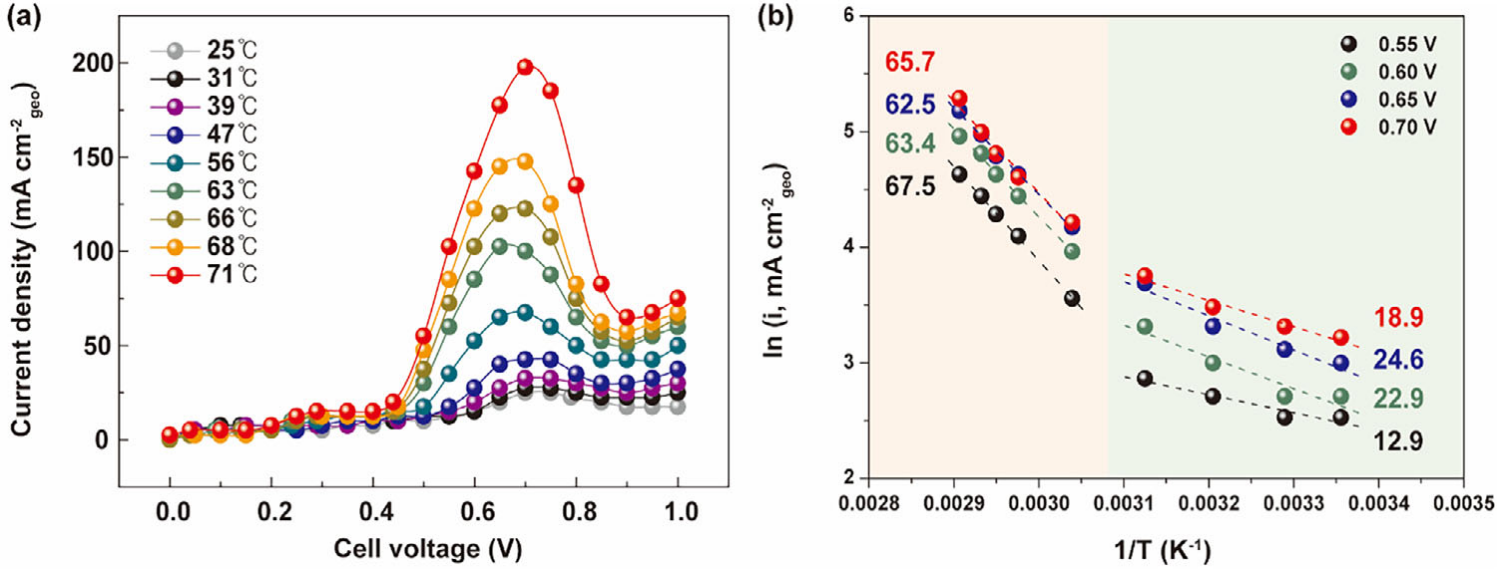
a) Polarization curves at various temperatures under 8 m KOH and 1 m NH_3_ concentrations and b) the Arrhenius plots at various potentials.

The findings from the NH_3_ concentration experiment in Figure 5 highlight the critical role of operating temperature in determining cell performance. To thoroughly investigate the temperature effect, we conducted AEC performance tests across various temperatures. A direct correlation was observed between increased temperature and enhanced AEC performance, with the rate of improvement notably surpassing that of other parameters (**Figure** **6**a).

This phenomenon can be attributed to three main factors: (1) ohmic resistance, (2) charge transfer resistance, and (3) mass transport resistance. In terms of ohmic resistance, a significant reduction in electrolyte resistance is observed (Equation (17)):

(17) $$\;I_{\mathrm{electrolyte}}=\frac{Il}{Ak}=\frac{jl}{k}\;$$

The ion conductivity of the electrolyte increases with temperature, thereby reducing its resistance and enhancing cell performance. Second, with respect to charge transfer resistance, the reaction rate constant increases with temperature, as described by the Arrhenius equation:

(18) $$k=Ae^{-\frac{E_{A}}{RT}}$$

Increasing temperature accelerates the AOR and HER reaction rates, resulting in higher current densities. Lastly, regarding mass transport resistance, ion movement within the electrolyte increases with increasing temperature. Notably, a significant increase in current is observed above 70 °C, likely caused by the electrolyte reaching its boiling point at elevated temperatures, leading to the formation of bubbles. As the ammonia transitions from liquid to gas, a two‐phase flow develops, affecting the flow field and the porous structure of the electrode layers.^[^ ^11c^ ^]^ This change facilitates the transfer of ammonia to the reaction sites in both liquid and gaseous phases, significantly enhancing mass transport efficiency and increasing current output. Furthermore, we detected a significant transition in the reaction mechanism between 50 °C and 70 °C, as demonstrated by the correlation between temperature and current density. Figure 6b illustrates the Arrhenius plots at various potentials, with the x‐axis representing the inverse of temperature (1/T) and the y‐axis showing the natural logarithm of the current density. The activation energy can be derived from the slopes of these Arrhenius plots. Notably, a significant shift in activation energy values begins around 55 °C. Specifically, the activation energy (E_a_) increases more than 3.5 times compared to the lower temperature region, with E_a_ = 65.7 kJ mol^−1^ above 55 °C and E_a_ = 18.9 kJ mol^−1^ below 55 °C at 0.7 V. This change indicates a shift in the reaction mechanism from the G–M mechanism to the O–S mechanism. Similar trends were observed at 0.55, 0.6, and 0.65 V, supporting the hypothesis that a change in the dimerization step contributes to the mechanism shift.

This study aims to investigate variations in AEC performance under different operating conditions using full‐cell experiments. Therefore, eliminating the influence of the cathode electrode is impractical. However, given the high HER kinetics, its impact on the overall results is assumed to be minimal. To further investigate the influence of ammonia on the cathodic HER performance, we conducted the HER performance tests under the ammonia conditions (1 m KOH + 0.1 m NH_3_). As shown in Figure S8 (Supporting Information), the HER performance of Pt/C catalyst is minimally affected by the presence of ammonia, validating our initial assumption that cathodic part contribution to the overall results is negligible. Future research will focus on a more detailed physicochemical analysis of AOR behavior in response to temperature changes on the platinum surface, which is beyond the scope of this study.

### 2.6 Durability of Zirfon‐Based AEC

Based on optimized assembly and operating conditions, we compared the performance and durability of Zirfon‐based, AEM‐based, and unstackable H‐type AECs. Due to severe degradation of the backbone and side chains in high‐concentration KOH for AEM, experiments with the AEM‐based cell were conducted under conditions of 1 m KOH and 1 m NH_3_, consistent with conventional AEM‐based water electrolyzer conditions.^[^ ^28^ ^]^

**Figure 7:**
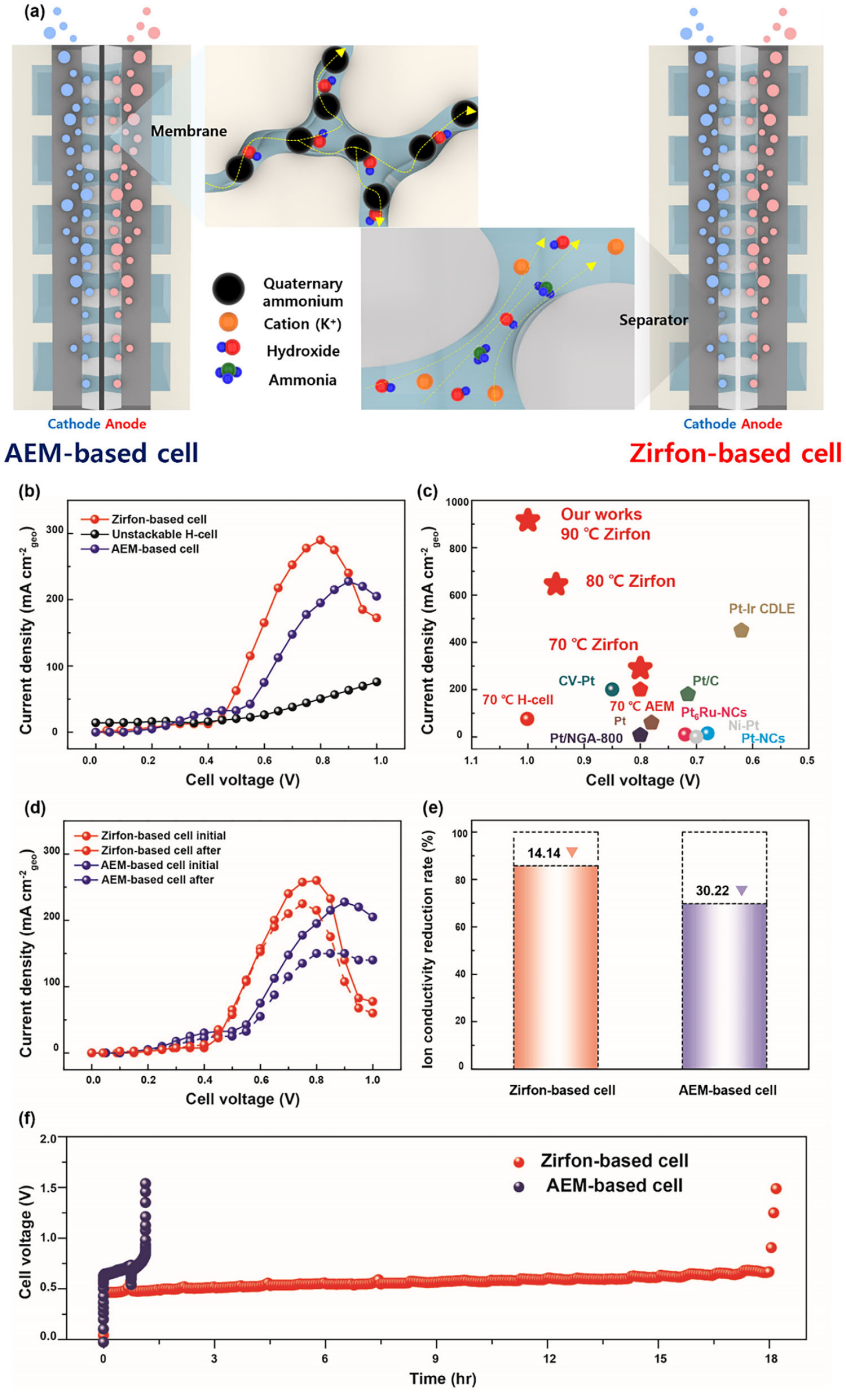
a) Schematic diagram of the Zirfon‐based and AEM‐based cells. The figure below the dotted line illustrates the separator and membrane during operation. b) Polarization curves for various cell types. c) Comparison of current density among different types of ammonia electrolysis cells. d) Changes in Zirfon and AEM ion conductivity before and after the durability test. e) Changes in the polarization curve of Zirfon and AEM before and after the durability test. f) Durability tests at fixed current density of 150 mA cm^−2.^

Generally, membranes and separators differ in their OH^–^ ion transport processes. In AEM, OH^–^ ion transport occurs through both the vehicle mechanism (standard diffusion) and the Grotthuss mechanism (ion hopping). The presence of rigid side chains creates areas of overlap, facilitating OH^–^ ion transport. Consequently, OH^−^ ions move through these limited overlapping regions, resulting in lower ion conductivity than the porous separator Zirfon.^[^ ^29^ ^]^ (**Figure** **7**a). This was directly confirmed by measuring the ion conductivity of Zirfon and AEM using the two‐probe method: Zirfon (8 m KOH + 1 mm NH_3_): 0.123 S cm^−1^, and AEM (1 m KOH + 1 m NH_3_): 0.0205 S cm^−1^. (Figures S9 and S10, Tables S1 and S2, Supporting Information)

Consequently, the high ion‐conductivity cell based on Zirfon exhibited superior performance, achieving a j_peak_ of 290 mA cm^−2^ at 0.8 V, as shown in Figure 7b. In contrast, the AEM‐based A and the unstackable H‐type cell, due to their low ion conductivities and high ohmic resistances, showed reduced peak current densities of 227.5 mA cm^−2^ at 0.9 V and 75.8 mA cm^−2^ at 1 V, respectively. These results demonstrate the superior performance of the Zirfon‐based cell. Furthermore, we conducted additional performance tests with the Zirfon‐based cell at higher temperatures of 80 °C and 90 °C. Notably, we achieved a j_peak_ of 645 mA cm^−2^ @ 0.8 V under 80 °C and 915 mA cm^−2^ @ 1.0 V under 90 °C, almost 2 and 3 times higher than the performance observed at 70 °C, respectively. (Figure S11, Supporting Information) The j_peak_ of the Zirfon‐based AEC is substantially higher than that reported for other AECs,^[^ ^8^, ^10^, ^11^, ^12^, ^30^ ^]^ demonstrating its remarkable performance. (Figure 7c; Table S3, Supporting Information). We firmly believe that applying advanced electrode fabrication methods, such as incorporating Ir into Pt and adjustments to the coating layer, could lead to substantial improvements in our system. Additionally, in Figure S12 (Supporting Information), a 2‐stack experiment confirmed the scalability of the system for large‐scale hydrogen production using the Zirfon‐based AEC.

Utilizing Zirfon offers significant economic advantages compared to the MEA‐based cell. In terms of cost‐effectiveness, most cell components, such as the end plates, current collectors, bipolar plates, and gaskets, remain consistent in different AEC designs. The main cost differences arise from the choice of separator and electrolyte. Zirfon UTP 500+, priced at approximately $ 167 m^−2^ (Agfa, 2021),^[^ ^31^ ^]^ is significantly more affordable than anion exchange membranes like FAA‐3‐50, which cost seven times more ($1250 m^−2^).^[^ ^32^ ^]^ Although the Zirfon‐based cell requires a higher concentration of KOH, the cost of KOH, at approximately $0.8 kg^−1^,^[^ ^33^ ^]^ is relatively low. Therefore, the additional electrolyte cost can be definitely compensated by the cost savings from using a Zirfon separator. Considering these factors, the overall system cost is more favorable when using Zirfon. While the operating conditions may involve higher KOH consumption, the savings in membrane costs make this approach commercially viable.

Furthermore, the Zirfon‐based AEC shows significant durability as well as performance, as shown in Figure 7d. With an accelerated durability test involving 100 repeated polarization curves from 0 to 1 V, the j_peak_ of the Zirfon‐based cell decreased by only 12.6% (from j_peak _initial_ = 257.5 mA cm^−2^ to j_peak _final_ = 225 mA cm^−2^), which is 34.1% lower than the AEM‐based cell (from j_peak _initial_ = 227.5 mA cm^−2^ to j_peak _final_ = 150 mA cm^−2^). This result correlates directly with the degradation of Zirfon and AEM. After the accelerated durability test, Zirfon showed a 14.1% decrease in ion conductivity, while AEM showed a 30.2% decrease. (Figure 7e) This degradation is attributable to the breakdown of the backbone and functional groups of AEM under KOH and NH_3_ conditions. The high durability of the Zirfon‐based AEC was observed under various KOH and NH_3_ electrolyte conditions, demonstrating that the Zirfon separator is more suitable for high‐performance AEC operation (Figures S9 and S10, Tables S1 and S2, Supporting Information).

The durability of the electrolytic cell under fixed conditions (voltage or current) is also essential. To address this, we conducted additional durability tests at a fixed current density of 150 mA cm^−2^ at 80 °C. (Figure 7f) The cell voltage of AEM‐based cell initially remained stable at around 0.65 V, but a significant performance degradation was observed after 70 minutes. In contrast, the cell voltage of Zirfon‐based AEC remained stable at around 0.55 V for over 18 hours, though a noticeable voltage increase was observed after this period, likely due to ammonia poisoning of the catalytic surface.^[^ ^34^ ^]^

Indeed, ammonia poisoning of the catalyst surface remains a persistent challenge. To mitigate this, periodic reducing potential pulses have been proposed as an effective strategy to reactivate the catalytic surface.^[^ ^35^ ^]^ This method helps desorb poisoning species (e.g., N_ads_) from the Pt surface, thereby improving long‐term cell performance. We conducted an additional long‐term test using a reactivation protocol, consisting of two steps: (1) 2 minutes of operation at 0.6 V, followed by (2) 1 minute at 0.0 V to the cell. Without the reducing potential pulses, the ammonia electrolysis cell degraded after 12 hours at 0.6 V. However, with the periodic pulses, the average output current per step was retained at ≈56% at 0.6 V over 100 hours, demonstrating the durability of Zirfon‐based AECs. (Figure S13, Supporting Information)

These experimental results suggest that long‐term operation of the Zirfon‐based AEC can be sustained by minimizing catalyst poisoning. We firmly believe that there is significant potential for achieving extended operation without ammonia poisoning through appropriate material and systematic approaches, however, addressing these approaches lies beyond the scope of this work.

To further investigate the morphology of the separator/catalyst interface before and after the long‐term test, we conducted SEM analysis (Figures S14 and S15, Supporting Information). The SEM images showed no significant changes after the long‐term test. Additionally, EDS data confirmed the absence of any Pt signal on the anode side of the Zirfon separator. ICP‐MS analysis revealed that the concentration of the dissolved Pt was found to be 81.44 µg cm^−2^, a negligible amount, supporting the durability of the Zirfon‐based ammonia electrolysis cell.

## 3 Conclusion

In this study, we introduced a novel Zirfon‐based AEC system for the first time and systematically optimized its operational parameters. By precisely adjusting the assembly and operating conditions, particularly the cell clamping pressure and electrolyte flow rate, we attained optimal performance with a cell clamping pressure of 100 kgf cm^−2^ and an electrolyte flow rate of 200 cc/min in our AEC. Additionally, our investigation of the variations in electrolyte concentration with temperature provided insights into the changes in the AOR mechanism depending on the operating temperature. Under these optimized conditions, our cell tests exhibited a significant enhancement in AEC performance at the system level, achieving a maximum current density of 915 mA cm^−2^. This performance is approximately four times greater than that of a non‐stackable H‐type cell and exceeds those reported for other H‐type cells and AEM‐based cells. Furthermore, the Zirfon separator exhibited a lower rate of conductivity loss compared to AEMs during cycling tests, with a reduction of 14.1% for Zirfon versus 30.2% for AEMs, demonstrating the superior durability of the Zirfon‐based AEC. This study provides valuable insights into strategies for enhancing AEC performance from a systematic perspective, paving the way for further advancements in AEC technology.

## 4 Experimental Section

### Material

The chemicals used include ammonium hydroxide solution (NH_4_OH, 6 m, Daejung), potassium hydroxide solution (KOH, 45 wt.%, Daejung), Nafion ionomer solution (5 wt.%, Sigma), isopropyl alcohol (IPA, Sigma), commercial Pt/C (46.9 wt.%, Tanaka), commercial Ir/C (40 wt.%, Premetek Co), commercial Pd/C (20 wt.%, Premetek Co), ethyl alcohol (99.9%, Daejung), hydrochloric acid (HCl, 35 wt,%, Daejung), anion exchange membrane (FAA‐3‐50, Fuel Cell Store), and Zirfon (27 × 27 mm^2^, Yulim Engineering)

### Preparation of Electrodes

Nickel foam (Dongjin Metaltech, square shape, 2 × 2 cm^2^, 1.6 mm thickness, 95 pores per inch, density: 420 g m^−2^) was used as the conductive support for the electrode and the porous transport layer (PTL). To remove the surface oxide layer, the nickel foam was cleaned through sequential sonication in the following steps: 1) 10 mL deionized water (DI water, 18.2 MΩ·cm) with 5 mL HCl, 2) DI water, and 3) ethyl alcohol, each for 10 min. Subsequently, catalyst ink was applied to the nickel foam. This ink consisted of 0.15 g catalyst powder, 1.5 mL DI water (18.2 MΩ·cm), 2.7 mL IPA, and 0.318 mL Nafion ionomer solution. The ink was sonicated for 30 min and uniformly dispersed onto the nickel foam surface using a spray gun. It was then dried at 60 °C for 30 min. The catalyst loading was ≈4 mg_pt_ cm^−2^.

### Ammonia Electrolysis Cell Test

The electrolyte temperature was maintained using a heating mantle (Misung) and a thermocouple. The flow rate at the electrode was regulated by a peristaltic pump (Dongbang Hitech), ranging from 50 to 250 cc min^−1^. The cell clamping pressure was adjusted between 60 to 120 kgf cm^−2^. Performance tests for the AEC were conducted using a DC power supply (ODA Technologies). Current density output was measured by increasing the voltage by 0.05 V. All cell tests were conducted after a thorough activation process was completed. Traditional alkaline electrolysis was used to compare the Zirfon‐based cell with the interelectrode gap maintained at 5 mm. Specific experimental conditions are detailed in each respective section.

### Electrochemical Durability Test

Electrochemical durability tests were conducted using chronopotentiometry (CP, at 150 mA cm^−2^) with a potentiostat (Metrohm Autolab, PGSTAT204). To prevent the performance degradation of the ammonia electrolyzers due to the decrease in ammonia concentration, the electrolyte was replaced every 45 minutes. The metal contents dissolved in the electrolyte during these tests were analyzed using ICP‐MS.

### Durability Tests with Reactivation Pulse Protocol

Durability tests with reactivation pulse protocol were conducted using DC power supply. The cell was operated following a two‐step protocol: (1) 2 minutes of operation (start‐up) at 0.6 V, followed by (2) 1 minute at 0.0 V to the cell (shut‐down). To prevent the performance degradation of the ammonia electrolyzers due to the decrease in ammonia concentration, continuous ammonia gas purging was conducted. The ammonia gas consists of 20% NH₃ and 80% nitrogen.

### Ion Conductivity

Ion conductivity measurements of the membranes were performed using the two‐probe resistance measurement technique with electrochemical impedance spectroscopy, employing a potentiostat (PGSTAT204, Metrohm) at 80 °C and various electrolyte concentrations. Impedance was measured at frequencies ranging from 0.01 to 10 kHz. The ion conductivity of the samples was calculated using the following equation (Equation (19)):

(19) $$\mathrm{Ion}\;\mathrm{conductivity},\sigma \left(S/\mathrm{cm}\right)=\frac{l}{RS}\;$$

where *R* is the membrane's ohmic resistance, *l* is the distance between electrodes, and *S* is the active area of the membrane.^[^ ^36^ ^]^

### Inductively Coupled Plasma Mass Spectroscopy (ICP‐MS)

The metal concentrations in the fabricated sample or electrolyte were analyzed using ICP‐MS (PerkinElmer, NexION 300s).

### Field Emission Scanning Electron Microscopy (FE‐SEM)

The morphology of the fabricated samples was examined using FE‐SEM (Zeiss, SUPRA25) at an accelerating voltage of 10 kV.

## Conflict of Interest

The authors declare no conflict of interest.

## Supporting information



Supporting Information

## Data Availability

The data that support the findings of this study are available from the corresponding author upon reasonable request.
