# Supplementary material for: Highly Efficient and Durable Ammonia Electrolysis Cell Using Zirfon Separator
Source: Adv Sci (Weinh). 2025 Jan 31;12(12):2500579. doi: 10.1002/advs.202500579 (PMC11947998; doi:10.1002/advs.202500579)
Supplement: Supplementary file 1 — Supporting Information [file ADVS-12-2500579-s001.docx]

Supporting Information

Highly Efficient and Durable Ammonia Electrolysis Cell using Zirfon Separator

Haeyong Shin ^1†^, Sang-Mun Jung ^1†^, Young Jin Lim^1^, O-Jung Yim^1^, Byung-Jo Lee^1^, Kyu-Su Kim^1^, In-Ho Baek^1^, Jinwoo Baek^1^, Jinhyeon Lee^1^, Yong-Tae Kim^1^*

^1^Department of Materials Science and Engineering, Pohang University of Science and Technology, Gyeongbuk 37673, Republic of Korea

^†^ H Shin and S-M Jung contributed equally to this work.

E-mail: yongtae@postech.ac.kr


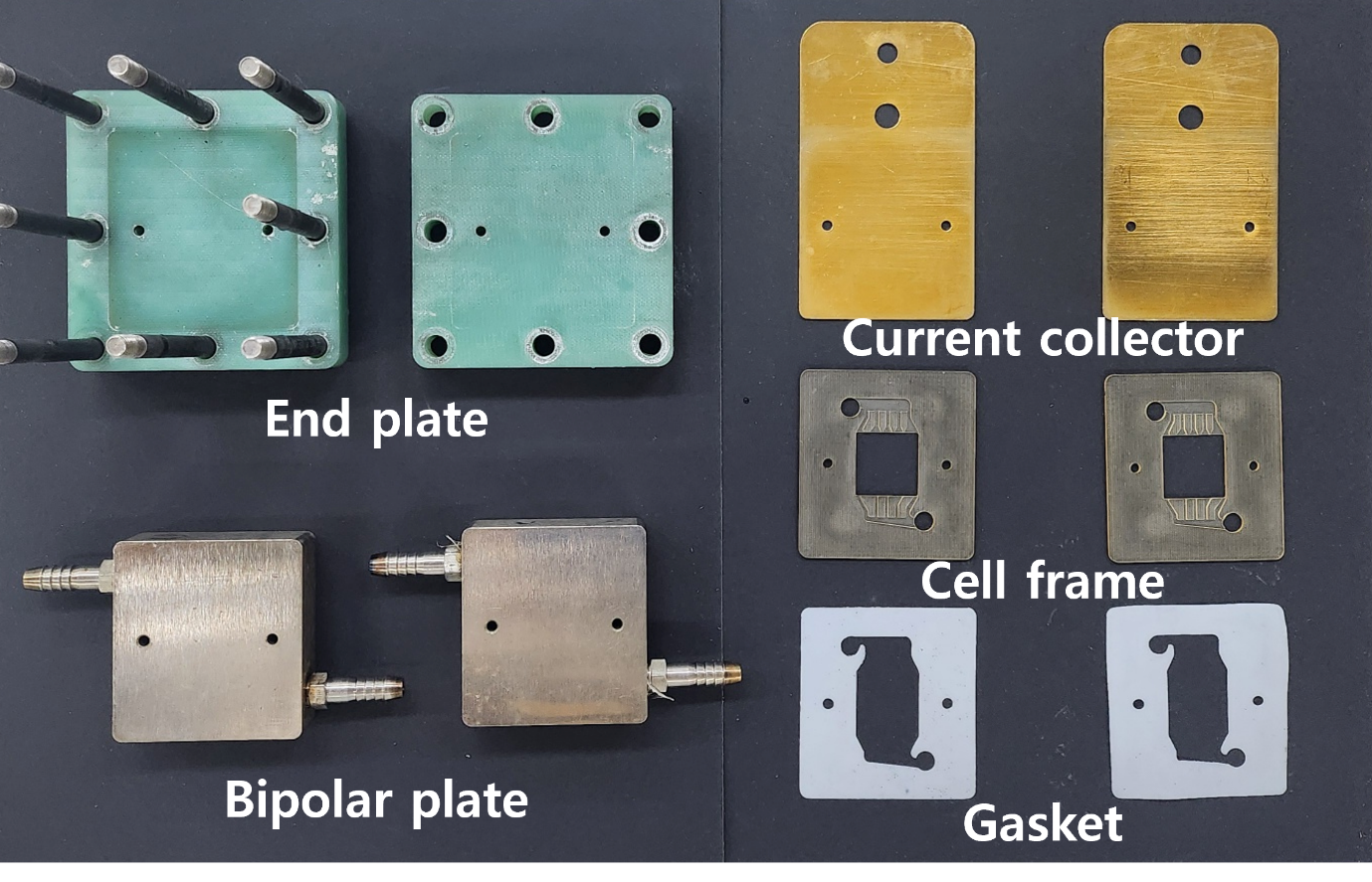


**Figure S1.** Photo of the ammonia electrolysis cell component.


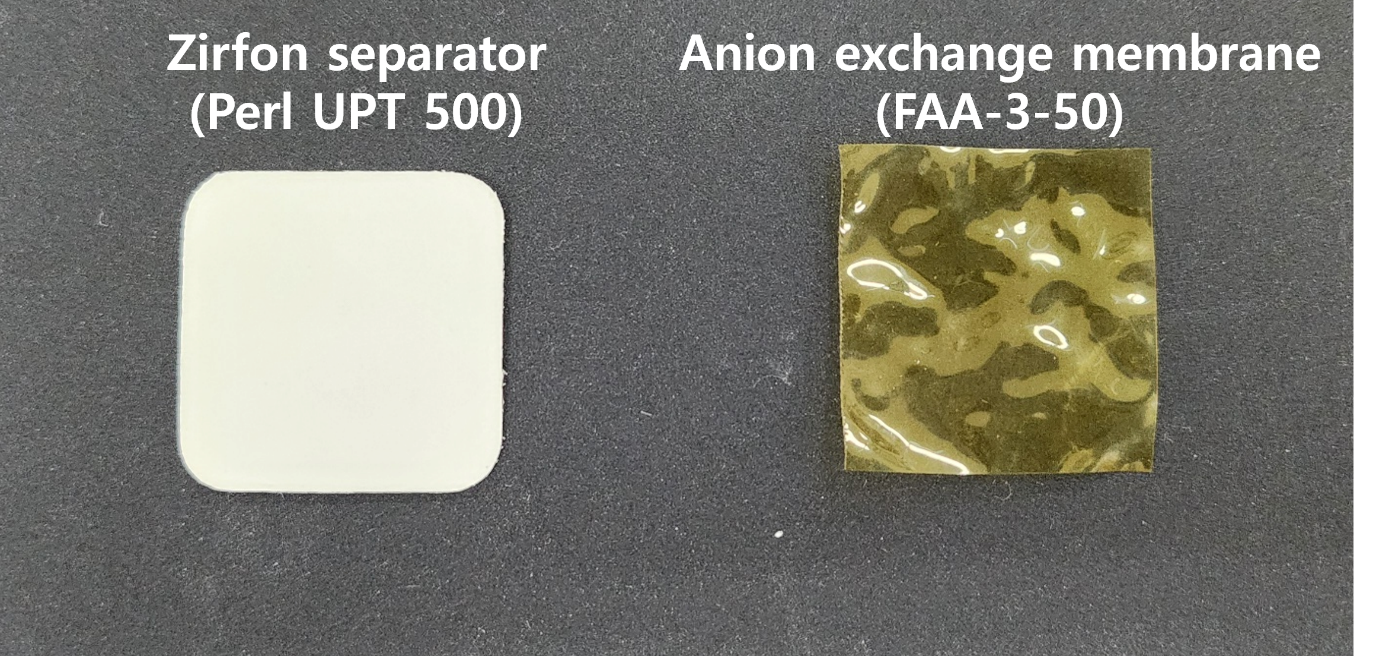


**Figure S2.** Photo of the zirfon separator (Perl UPT 500) and anion exchange membrane (FAA-3-50).


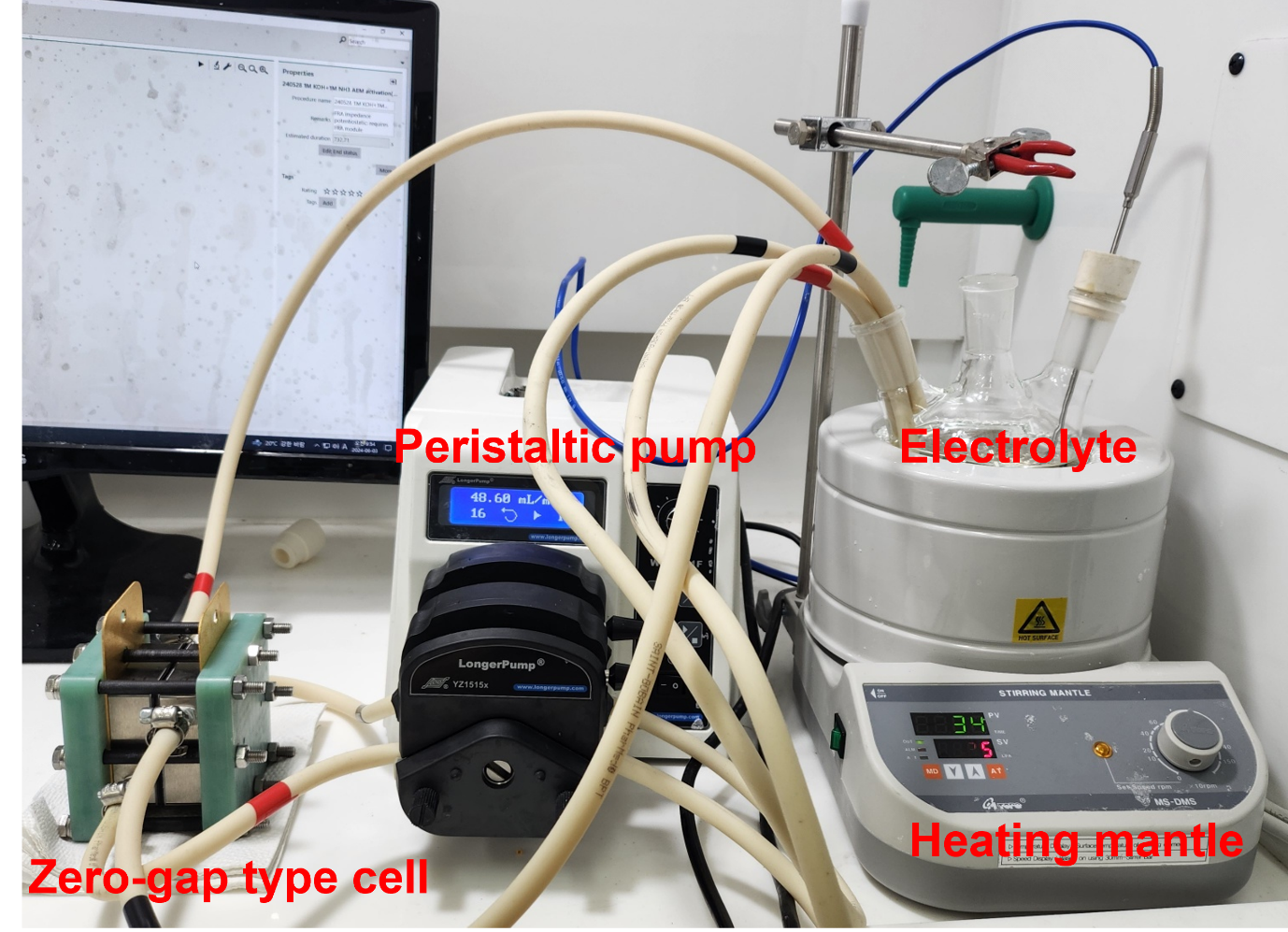
**Figure S3.** **Photo of the operation of the ammonia electrolysis cell**


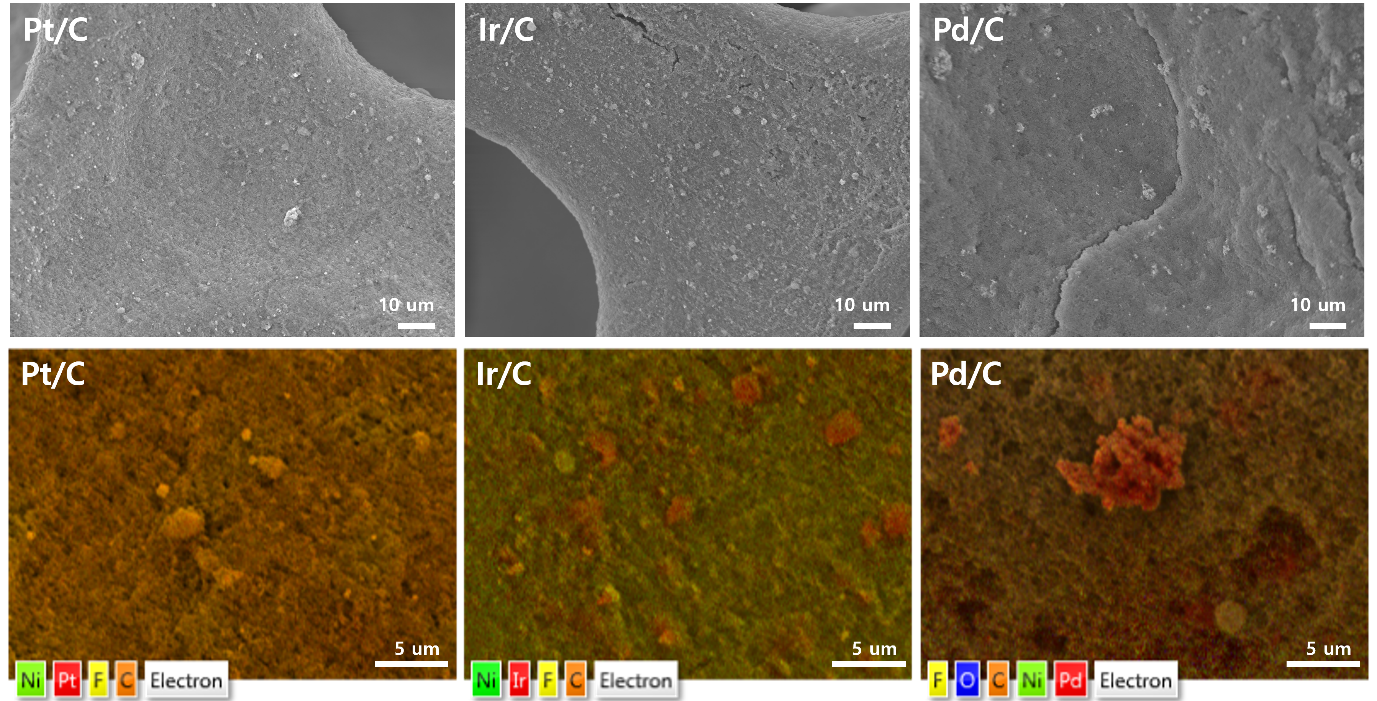
**Figure S4.** Scanning electron microscopy (SEM) and Energy-dispersive X-ray spectroscopy (EDS) images with nanoparticle dispersed on Ni foam electrodes.

**Figure S5.** AEC polarization curves at different metal anodes.


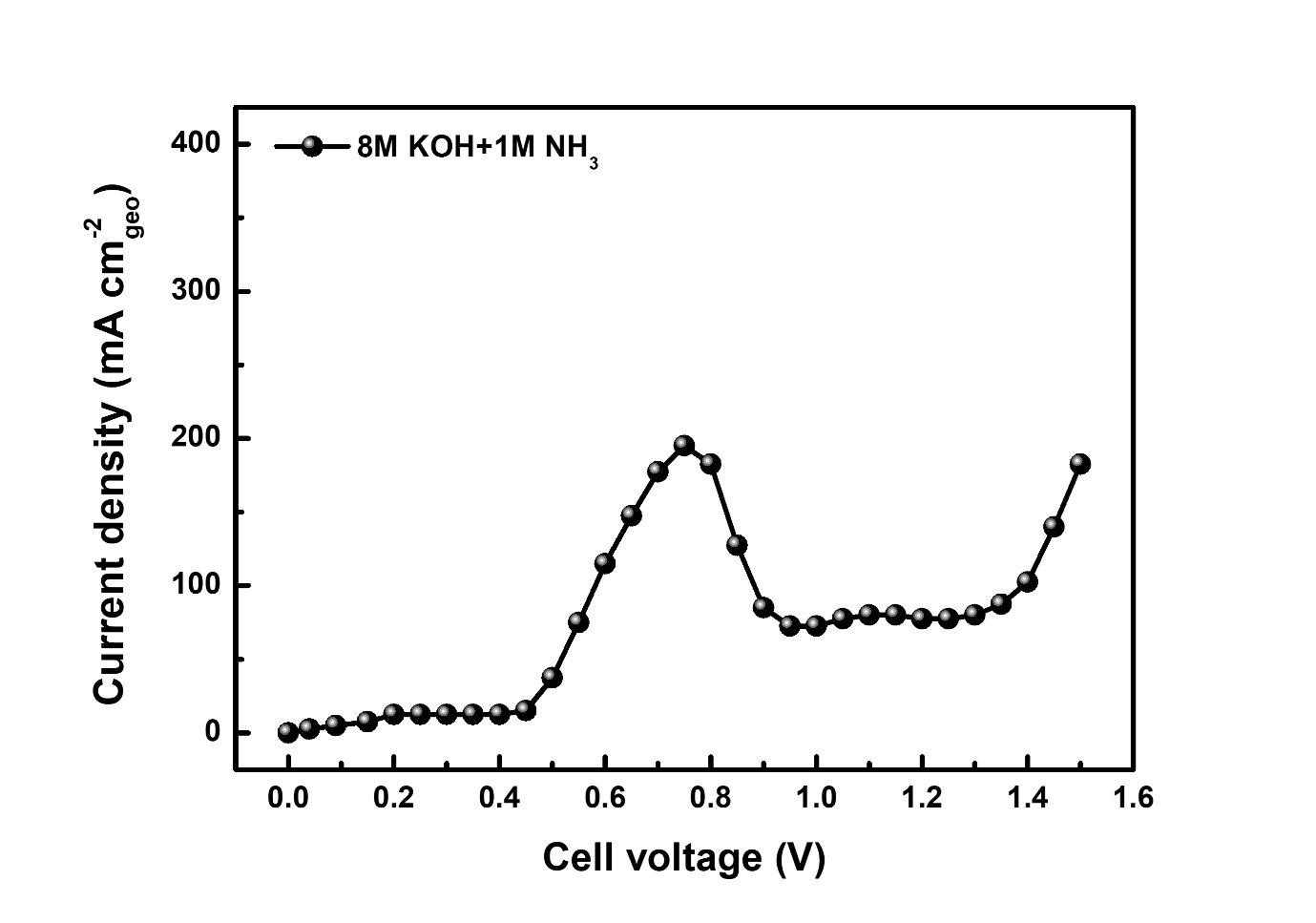
**Figure S6.** AEC polarization curved under 8 M KOH containing 1 M ammonia solution. Conducted measurements within the range of 0 V to 1.5 V.


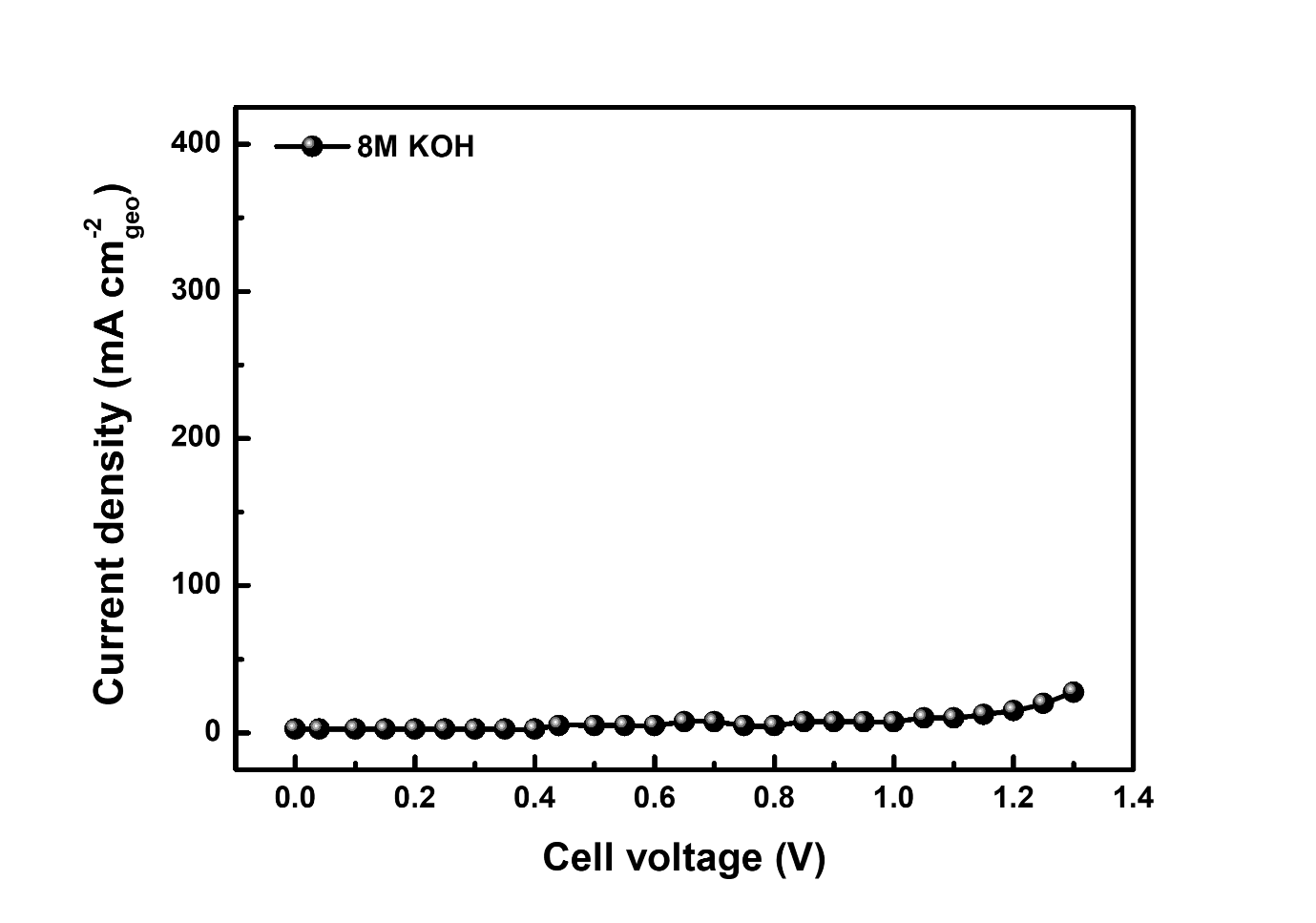
**Figure S7.** Polarization curve in 8 M KOH. Conducted measurements within the range of 0 V to 1.3 V.


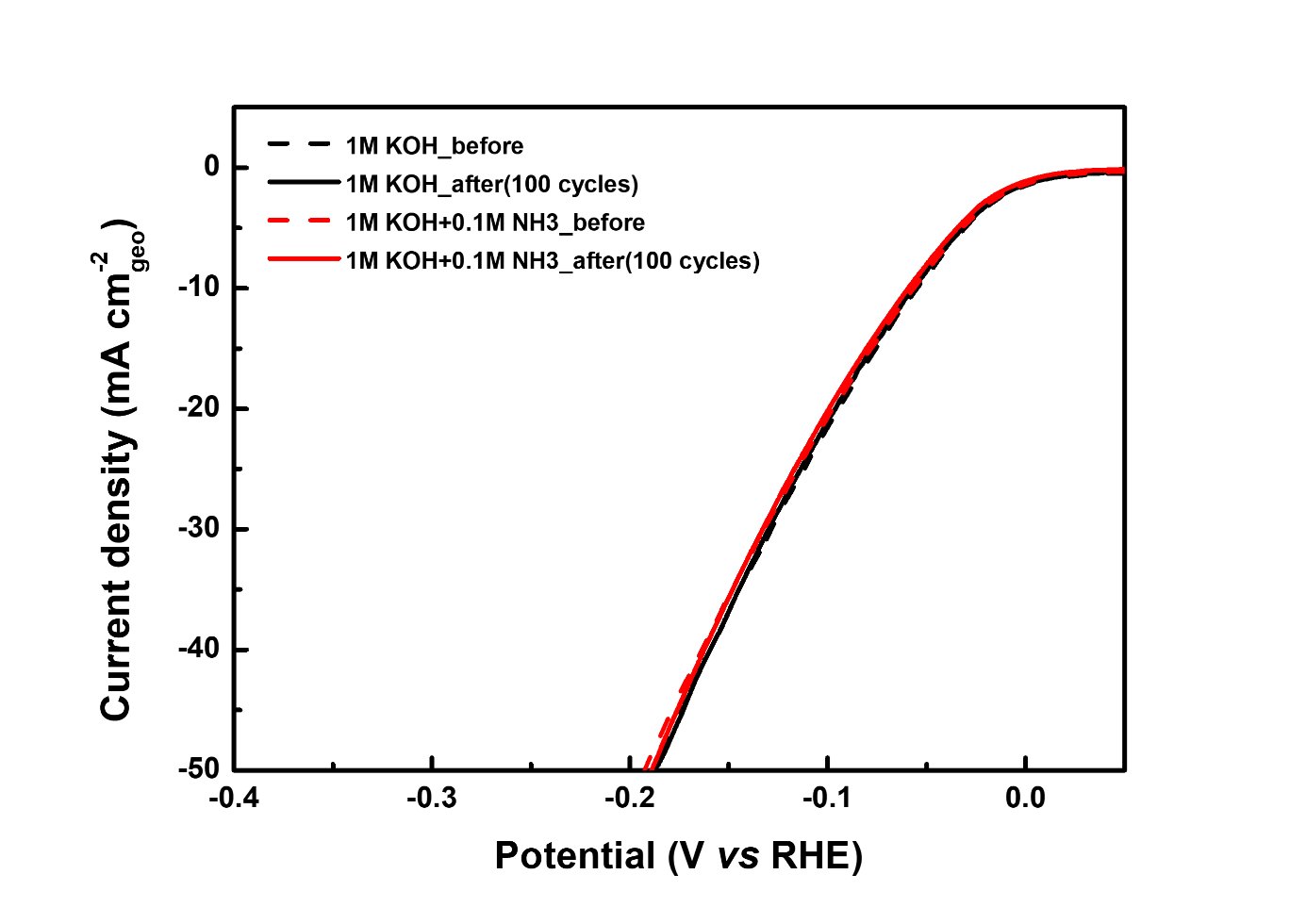


**Figure S8.** HER performance tests under the ammonia conditions (1M KOH + 0.1M NH_3_).

**
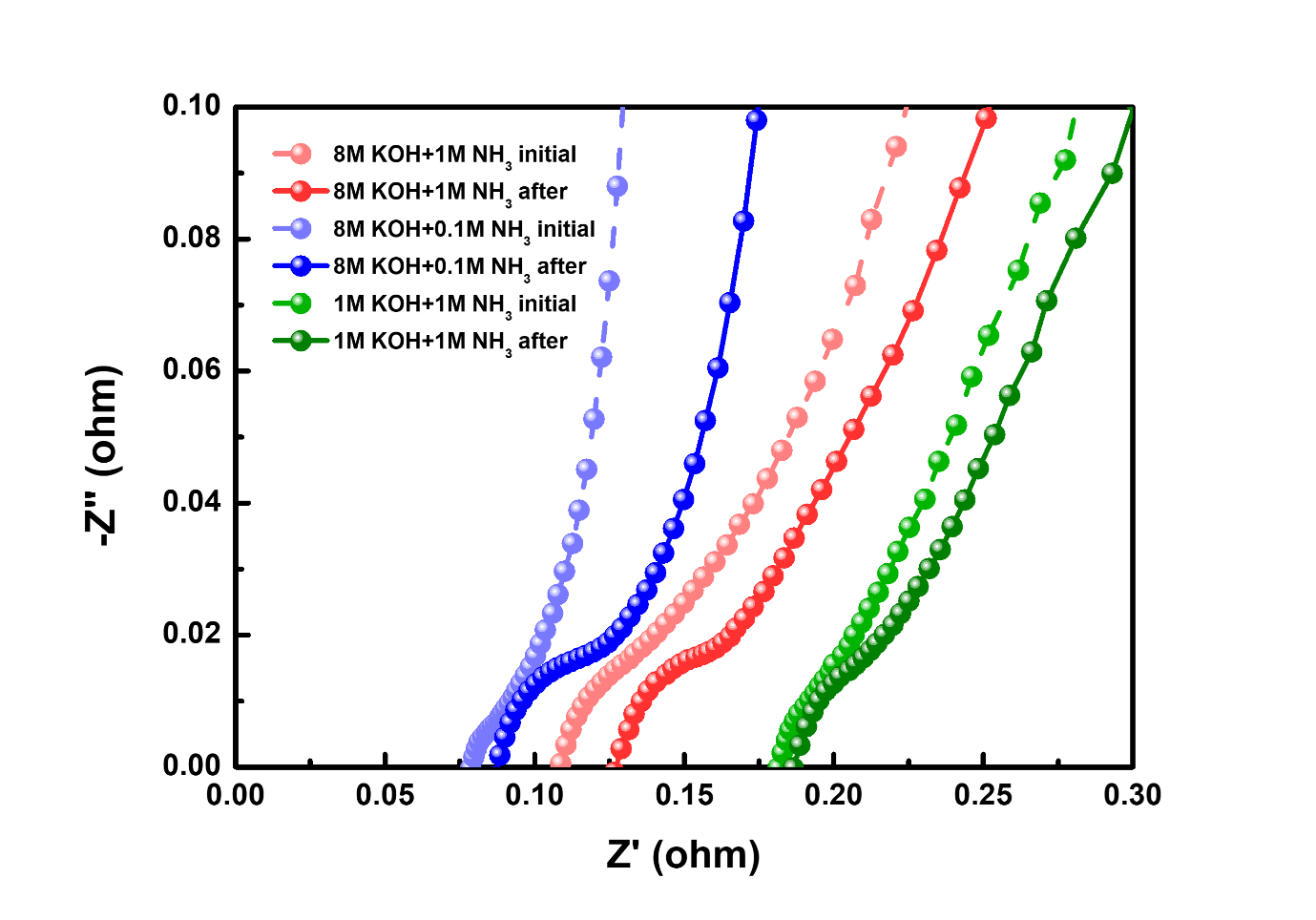
Figure S9.** Electrochemical impedance spectroscopy (EIS) measured before and after durability tests under various electrolyte concentrations using a Zirfon separator.

**
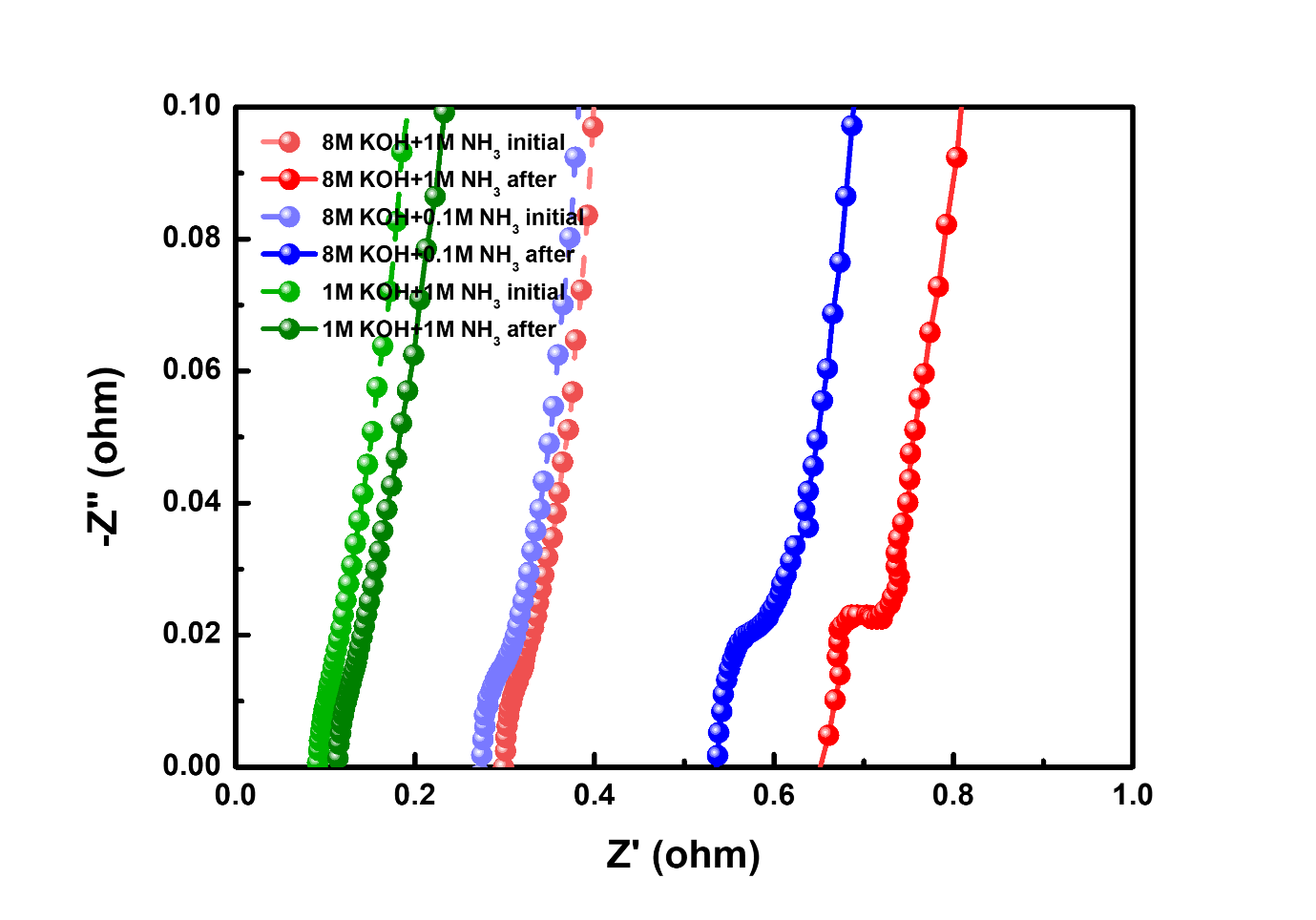
Figure S10.** Electrochemical impedance spectroscopy (EIS) measured before and after durability tests under various electrolyte concentrations using a AEM membrane.


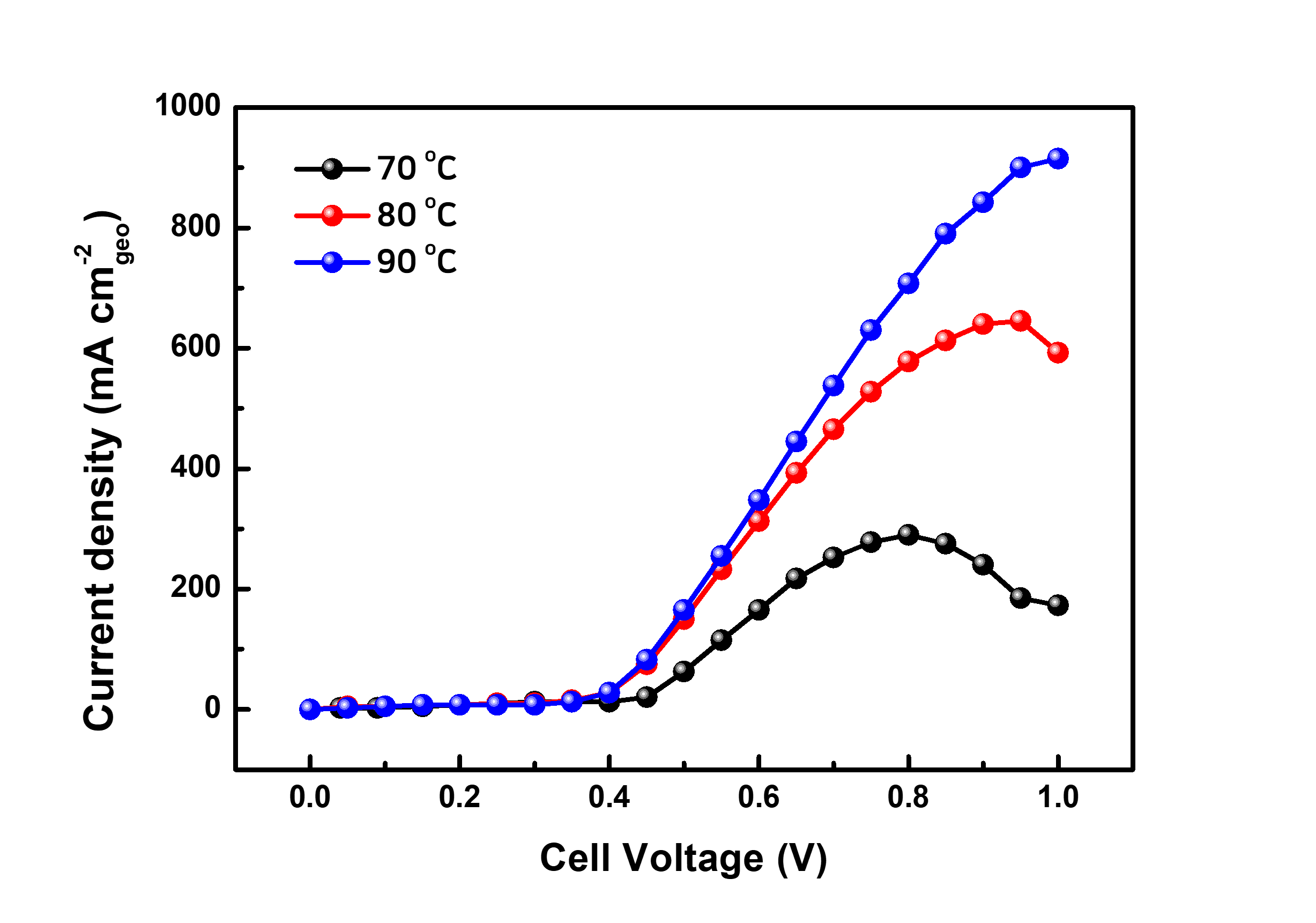


**Figure S11.** Polarization curves of Zirfon-based AEC at 70, 80 and 90 °C.

**Note:** A significant increase in current density is observed above 70°C, likely caused by the electrolyte reaching its boiling point at elevated temperatures, leading to the formation of bubbles. As the ammonia transitions from liquid to gas, a two-phase flow develops, affecting the flow field and the porous structure of the electrode layers. This change facilitates the transfer of ammonia to the reaction sites in both liquid and gaseous phases, significantly enhancing mass transport efficiency and increasing current output.

**
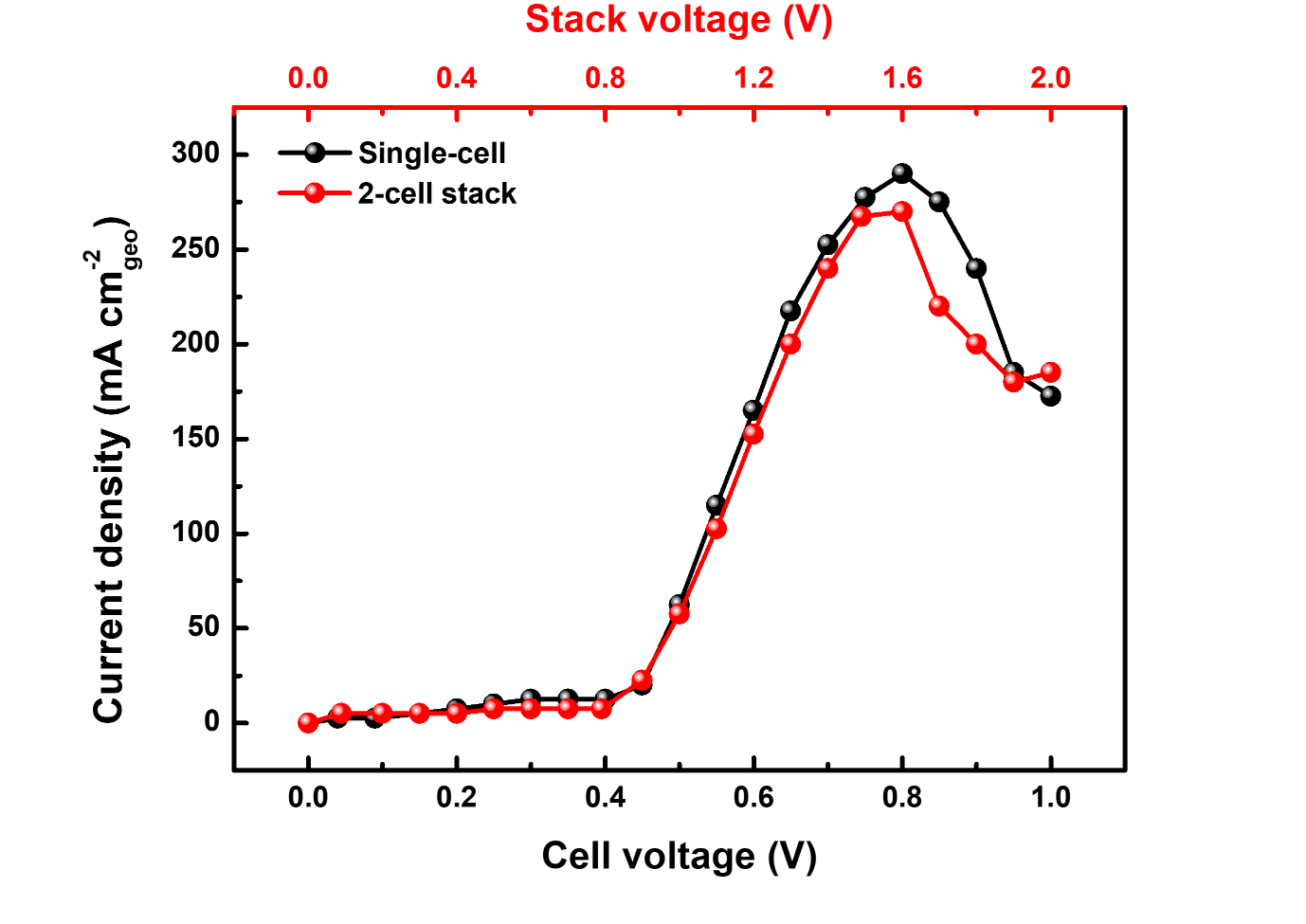
Figure S12.** Polarization curve of a 2-stack cell utilizing a zirfon separator.


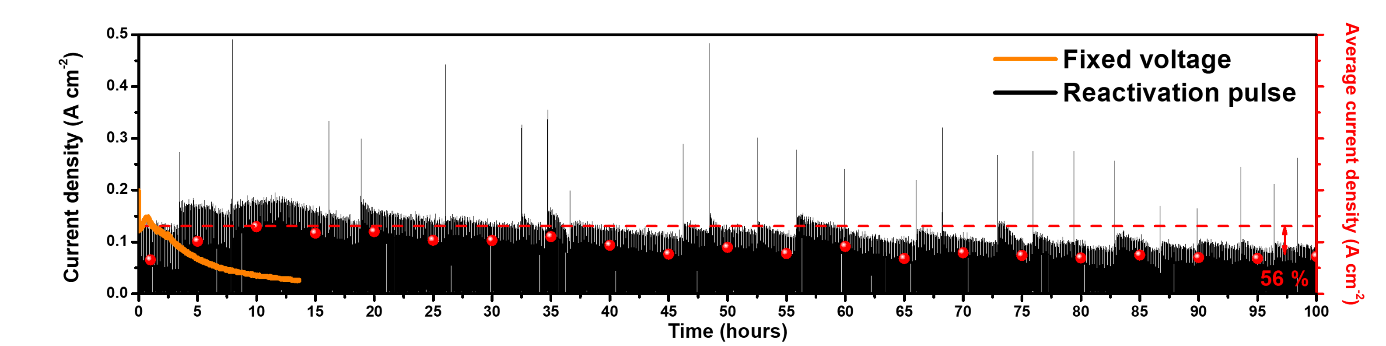


**Figure S13.** Durability tests with reactivation pulse protocol at fixed voltage 0.6V.

**Figure S14.** The SEM and EDS results of Zirfon before the long-term test. To obtain higher resolution images, gold sputtering was applied prior to the measurements.


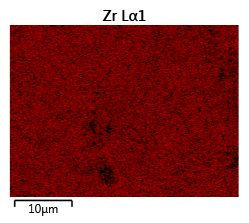

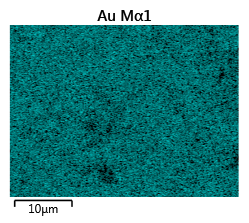

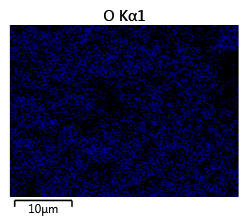

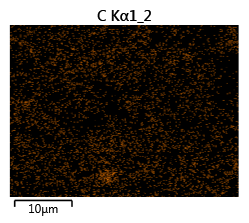

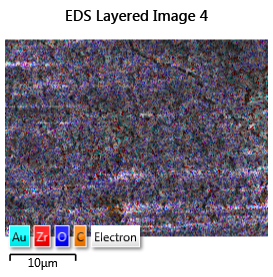


**Before**


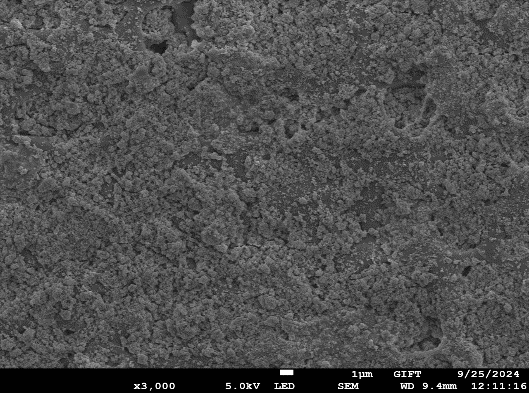


**Before**


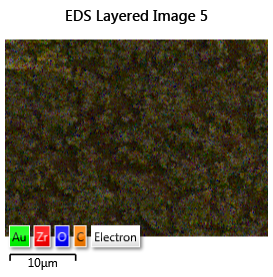

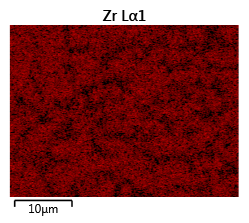

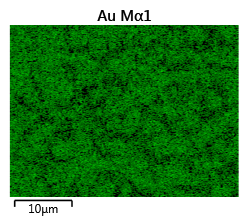

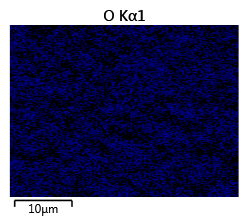

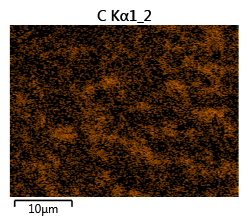


**After**


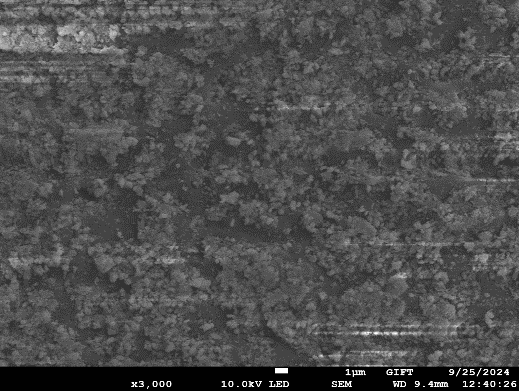


**After**

**Figure S15.** The SEM and EDS results of Zirfon after the long-term test. To obtain higher resolution images, gold sputtering was applied prior to the measurements. The analysis confirmed that no Pt was attached to the surface of Zirfon before or after the test.

**Table S1.** Changes in ion conductivity before and after durability tests under various electrolyte conditions using a Zirfon separator.


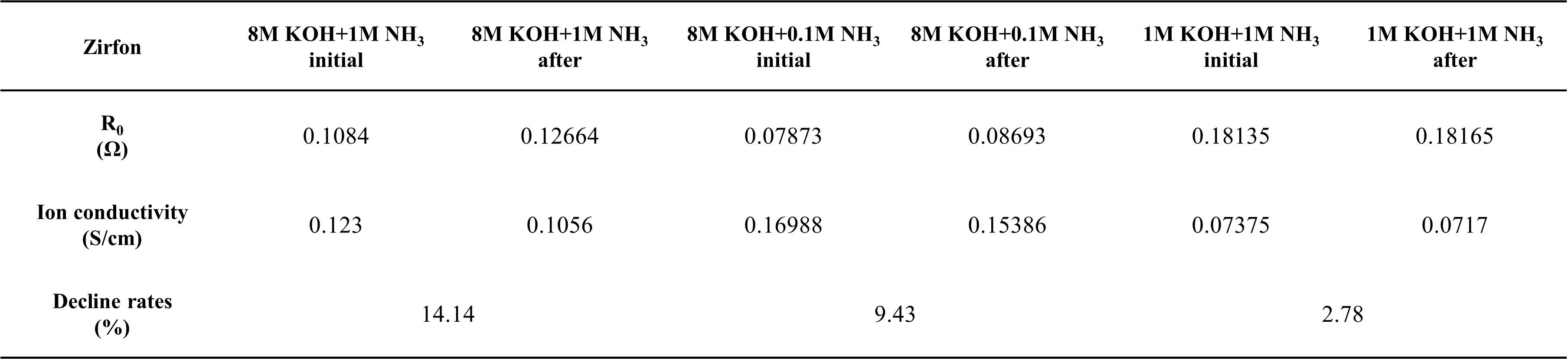


**Table S2.** Changes in ion conductivity before and after durability tests under various electrolyte conditions using a AEM membrane.

**
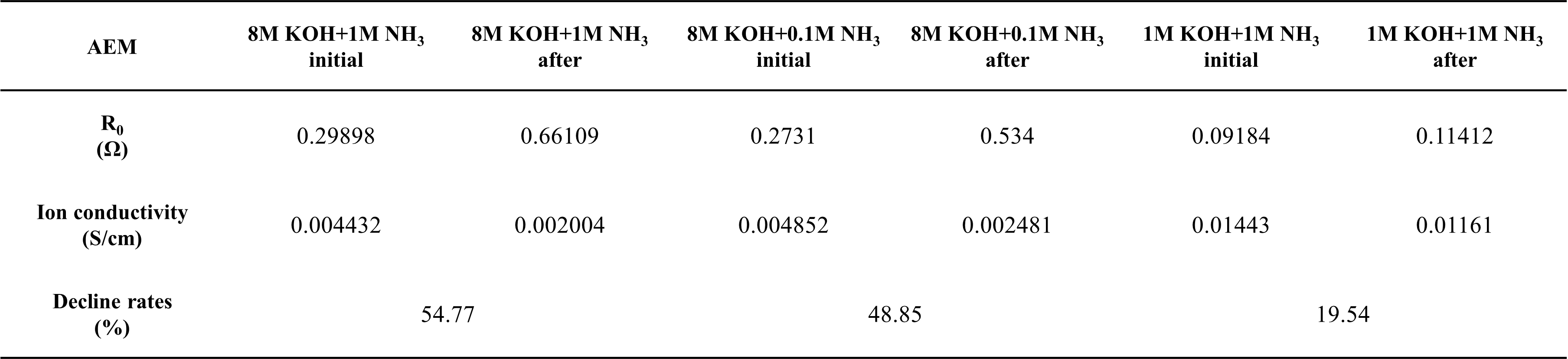
**

**Table S3.** Summary of AEC condition and performance.

| **Anode**  **(Loading/**  **mg cm^-2^_geo_)** | **Cathode**  **(Loading**  **/mg cm^-2^_geo_)** | **Cell-type** | **Membrane** | **Cell voltage**  **(V_cell_)** | **Electrolyte** | **T (℃)** | **Refs.** |
| --- | --- | --- | --- | --- | --- | --- | --- |
| Pt-NCs | Pt-NCs | Conventional  cell | - | 0.68 V/ 15  mA cm^-2^ | 0.1 M NH_4_OH | 25 | ^1^ |
| CV-Pt | Pt wire | Conventional  cell | - | 0.85 V/ 200  mA cm^-2^ | 1 M NH_3_ | 60 | ^2^ |
| Pt_6_Ru-NCs | Pt_6_Ru-NCs | Conventional  cell | - | 0.72 V/ 10  mA cm^-2^ | 0.1 M NH_3_+1 M  KOH | - | ^3^ |
| Ni-Pt | Pt Plate | Conventional  cell | - | 0.7 V/ 0.132 E-3  mA cm^-2^ | 1 M NH_3_+1 M  KOH | 25 | ^4^ |
| Pt/NGA-800 | Pt/NGA-800 | MEA | PEM | 0.8 V/ 7.5  mA cm^-2^ | 0.1 M NH_3_+1 M  KOH | - | ^5^ |
| Pt/C | Pt/C | MEA | AEM:  A201 | 0.715 V/ 180  mA cm^-2^ | 2 M NH_3_+1 M  KOH | 60 | ^6^ |
| Pt | Pt-Ru foil | MEA | Polypropylene | 0.78 V/ 60  mA cm^-2^ | 1 M NH_3_+5 M  KOH | 60 | ^7^ |
| Pt-Ir CDLE | Pt/C | MEA | FAA-3-50 | 0.62 V/ 450  mA cm^-2^ | 2 M NH_3_+2 M  KOH | 80 | ^8^ |
| Pt/C | Pt/C | Zero-gap | Zirfon | 0.8 V/ 290  mA cm^-2^ | 1 M NH_3_+8 M  KOH | 60 | ^Our works^ |
| Pt/C | Pt/C | Zero-gap | Zirfon | 0.8 V/ 645  mA cm^-2^ | 1 M NH_3_+8 M  KOH | 70 | ^Our works^ |
| Pt/C | Pt/C | Zero-gap | Zirfon | 1 V/ 915  mA cm^-2^ | 1 M NH_3_+8 M  KOH | 80 | ^Our works^ |

**References**

[1] H.-Y. Sun, G.-R. Xu, F.-M. Li, Q.-L. Hong, P.-J. Jin, P. Chen, Y. Chen, *Journal of Energy Chemistry* **2020**, *47*, 234-240.

[2] Y. Yang, J. Kim, H. Jo, A. Seong, M. Lee, H.-K. Min, M.-g. Seo, Y. Choi, G. Kim, *Journal of Materials Chemistry A* **2021**, *9*, 11571-11579.

[3] J. Gwak, M. Choun, J. Lee, *ChemSusChem* **2016**, *9*, 403-408.

[4] Y. Zhou, G. Zhang, M. Yu, X. Wang, J. Lv, F. Yang, *ACS Sustainable Chemistry & Engineering* **2018**, *6*, 8437-8446.

[5] Q. Xue, Y. Zhao, J. Zhu, Y. Ding, T. Wang, H. Sun, F. Li, P. Chen, P. Jin, S. Yin, *Journal of Materials Chemistry A* **2021**, *9*, 8444-8451.

[6] K. Yao, Y. Cheng, *International journal of hydrogen energy* **2008**, *33*, 6681-6686.

[7] F. Vitse, M. Cooper, G. G. Botte, *Journal of Power Sources* **2005**, *142*, 18-26.

[8] D. Yoon, S. Chung, M. Choi, E. Yang, J. Lee, *Journal of Energy Chemistry* **2024**, *93*, 352-360.
